# A survey of adolescent experiences of human papillomavirus vaccination in the Manchester study

**DOI:** 10.1038/sj.bjc.6605362

**Published:** 2009-10-06

**Authors:** L Brabin, S A Roberts, R Stretch, D Baxter, P Elton, H Kitchener, R McCann

**Affiliations:** 1 Academic Unit of Obstetrics and Gynaecology, School of Cancer and Imaging Sciences, University of Manchester, Manchester, UK; 2 Health Methodology Research Group, School of Community Based Medicine, University of Manchester, Manchester, UK; 3 Public Health Department, Stockport Primary Care Trust, Stockport, UK; 4 Public Health Department, Bury Primary Care Trust, Bury, UK; 5 Greater Manchester Health Protection Unit, Eccles, UK

**Keywords:** HPV, adolescent girls, perceptions, side effects, sexual behaviour

## Abstract

**Background:**

There is little information on girls' experiences of human papillomavirus (HPV) vaccination in the prevention of cervical cancer. We investigated the views of adolescent girls who had been offered the vaccine as part of a feasibility study conducted in Manchester.

**Methods:**

All 12 to 13-year-old girls in two primary care trusts were offered three doses of Cervarix (manufactured by GlaxoSmithKline). A letter was sent to 1084 parents who had consented to research follow-up. It requested parents to pass a questionnaire regarding HPV vaccination to their daughters to complete and post back in a prepaid envelope.

**Results:**

A total of 553 girls completed the questionnaire. Altogether, 77% (422) had shared with their parents in the vaccine decision. In all, 42% (*n*=13) of girls, whose parents refused vaccination, stated that they wanted the vaccine, whereas 10% (50) of those who were vaccinated did not want the vaccine. Although 54% (277) said the vaccine was very important to them, 39% (153) of vaccinated girls thought they might not recommend it to others. The vaccine was perceived to be painful and there were exaggerated rumours of serious adverse events and needle scares. A total of 79% (420) of girls agreed with a statement that vaccination reminded them of the risks of sexual contact, but 14% (73) agreed they might take more sexual risks because they had been vaccinated.

**Conclusion:**

Girls of this age form their own views on HPV vaccination but parental support for vaccination remains important, especially for completing the three doses. By discussing the vaccine, parents can encourage their daughters to determine the importance and implications of HPV vaccination.

The Department of Health in England began routine vaccination of 12-year-old girls in September 2008, together with a catch-up programme for older teenagers. The programme aims at protecting adolescent girls against human papillomavirus (HPV) 16 and HPV 18, which are the types responsible for 70% of cervical cancer cases. Provisional uptake figures showing 86% coverage for the first of the three doses among 12-year olds is highly encouraging and suggest that the vaccine is acceptable to most adolescents and their parents (Department of Health, 2009a).

To date, there is almost no information on girls' experiences of HPV vaccination, particularly the views of younger adolescents, either from the United Kingdom or from other countries where the vaccine has been introduced. As parental consent is usually sought before vaccination, most research has focused on parental knowledge and attitudes associated with vaccine acceptance. In future, it will be relatively more important to understand the significance of HPV vaccination with regard to girls, as their health depends on actions and attitudes at the time of, and subsequent to, vaccination. For many girls, HPV vaccination will introduce the concept of possible exposure to a sexually transmitted infection and its consequences, the challenge of commitment to a three-dose vaccine schedule and their first active involvement in a preventive strategy that requires future follow-up actions – such as cervical screening or perhaps HPV testing – to ensure maximum protection against cervical cancer.

In 2007–2008, before the National Programme, we undertook a feasibility study offering bivalent HPV vaccination (Cervarix; GlaxoSmithKline, Rixensart, Belgium) to all Year-8 schoolgirls (aged 12 or 13 years) in two primary care trusts (PCTs) in Greater Manchester, UK (Brabin *et al*, 2008, Stretch *et al*, 2008a). In this school-based programme, uptake was 71% at the first dose, with 69% receiving the full three-dose course. This research provided a unique opportunity to investigate the views of adolescent girls who had been faced with an actual vaccine decision. In this paper, we describe the results of a post-vaccination questionnaire survey, the purpose of which was to understand the importance of HPV vaccination with regard to girls. We assessed adolescent acceptance of HPV vaccination and the role of girls in the decision-making process.

## Materials and methods

The North Manchester NHS Research Ethics Committee approved the study. Cervarix was offered at 0, 1 and 6 months to 2817 12 to 13-year olds between October 2007 and September 2008. A detailed description of vaccine delivery was published (Brabin *et al*, 2008, Stretch *et al*, 2008a). Essentially, each PCT was responsible for vaccine delivery but standardised, pretested information about the vaccine was provided by the research team in the form of an information sheet for parents and an educational film for girls (Vallely *et al*, 2008). Parents also received a flyer summarising the content of the educational film and details of parent evenings. School nurses who gave talks and answered questions showed the film in schools and at parent evenings. The information emphasised that the purpose of the vaccination was to prevent cervical cancer but was explicit with regard to the fact that HPV was sexually transmitted.

Parents completed a separate consent form agreeing for follow-up research questionnaires and the PCTs forwarded the names and addresses of consenting parents to the research team. As previously reported, 38% (1084) of the 2853 eligible parents agreed to be contacted, of whom 60% (651) completed a questionnaire (Parent Questionnaire 1) after Dose 1 (Stretch *et al*, 2008b). For this study, parental consent was required for the child to participate; hence, a short exit questionnaire (not reported here) was sent to eligible parents after Dose 3, with a letter requesting them to pass a separate questionnaire and a stamped, addressed envelope to their daughters. This questionnaire had been piloted and asked girls about their role in vaccine consent, factors that influenced their wish to be vaccinated and their perceptions of being vaccinated against a sexually transmitted infection.

Responses were mainly measured using Likert scales appropriate to the question asked. Proportions were summarised according to whether consent was given (‘consenters’) or refused (‘refusers’), and Fisher's exact tests were used to assess the significance of differences between groups. Missing responses are either reported as such or the proportions computed for responders, depending on the context of the specific question. The responses to an open question on rumours about HPV vaccination (‘Did you hear anything bad about the injection?’) were analysed semiqualitatively and illustrative comments are reported verbatim.

## Results

Of those parents who had agreed to be contacted, 52% (565) completed the exit questionnaire, as well as 51% (553) of their daughters. Among the girls returning questionnaires, 6% (33) of parents had refused vaccination; all but six of those consenting to vaccination had completed the three-dose schedule at that time.

### Factors influencing girls' attitudes to vaccination

#### Girls' participation in vaccine decision

In all, 77% (422) of girls stated that they had shared in the vaccine decision, parents decided for 19% (103) and 4% (25) made their own decision. Most girls (84%, *n*=459) said that parents listened to their views either ‘a lot’ or ‘quite a lot.’ A total of 42% (*n*=13) of girls whose parents refused vaccination stated that they wanted the vaccine, whereas 10% (50) of those who were vaccinated did not want the vaccine. Altogether, 70% (356) of vaccinated and 41% (13) of unvaccinated girls (*P*=0.0013) thought that girls of their age should be able to agree to have the vaccine without parental consent. Compared with 61% (20) of unvaccinated girls, 84% (433) of the vaccinated group (*P*=0.0012) said that they had received sufficient information, mainly from parents or at school. In all, 23% (127) sought additional information, mainly from the Internet (*n*=28).

### Importance accorded to HPV vaccination

The main reasons for girls consenting to vaccination were protection against cervical cancer (90.3%, *n*=465) and to avoid HPV infection (70%; *n*=361). Their views were influenced by parents (47%, *n*=241) and, to a lesser extent, by friends and school nurses (both 35%; *n*=178) or teachers (20%, *n*=105). A total of 54% (277) of girls considered HPV vaccination to be very important to them, and 39% (*n*=153) of vaccinated and 77% (*n*=24) of unvaccinated girls might not (‘probably’/‘no’) recommend vaccination to their peers in future.

#### Fear of vaccination

Rumours were reported by 49% (269) of girls. Almost a quarter of respondents (24%; *n*=132) had heard that the injection was painful and 9% (*n*=50) heard that it caused significant side effects, such as aching/swollen arms and fainting. Rumours of serious adverse events, such as allergic reactions, paralysis, jaundice, fits, cancer and warts, were reported by 8% (*n*=46), including death (16 of 46), as illustrated below:

- ‘My friend's mum said it has horrible side effects, like arthritis in the future.’
- ‘I heard it could leave you with brain damage.’
- ‘Someone told me she lost the use of her legs.’
- ‘One out of five girls can go in a toxic shock – that scared me a little.’
- ‘I heard that somebody died, but I think somebody made it up.’
- ‘Seven people died from the injection.’
- ‘People had died in America.’

Exaggerated needle scares were rife (3%; *n*=19), including descriptions of ‘*big*’ needles, double injections that got ‘*bigger each time*’, inoculation into the vein, hip or vagina and a report that the ‘*needle went right through the arm*,’ also misinformation such as:

‘Dose 1 injects cancer; Dose 2 and 3 take it away.’

Reproductive hazards were mentioned by 4% (*n*=20), such as infertility or congenital deformities (13 of 20). For example:

‘I heard that there was a possibility that your baby would come out deformed,’

and

‘It can cause demented babies.’

Rumours that the vaccine was ineffective (3%; *n*=19) and that girls were in a vaccine trial (4%; *n*=25) were also cited. Girls who had not been vaccinated reported fewer rumours (22 of 33; 33%) than did acceptors (245 of 516, 47%; *P*=0.047).

#### Vaccine experience

After vaccination, 20% (103) of girls reported feeling ill and 6% (30) wished to discontinue the course. Altogether, 5% (27) missed school during the course of vaccination, most (19) being absent for half a day or 1 day. The 11 girls who had sought medical advice at that time were all advised that the vaccine was unlikely to have caused their symptoms.

#### Sexual messages conveyed by HPV vaccination

Girls were asked to indicate their agreement (agree/disagree) with six statements that students had made during the piloting stages relating to how they, and others, perceived vaccination against a sexually transmitted infection (Table 1). The majority agreed that HPV vaccination made them think about their health and future sexual relationships. Nearly one-quarter found it embarrassing to be vaccinated against a sexually transmitted infection and would be hesitant to disclose this. Almost 14% (73) thought that being protected against HPV might lead them to take more sexual health risks in the future and 19% (99) said that boyfriends might expect them to.

## Discussion

The majority of girls in this study had talked to their parents and understood the purpose of vaccination. A substantial minority reported rumours of serious side effects but the main concern of girls was the pain of vaccination. This, and fear of needles, probably explains why many would not advise their peers to be vaccinated. A fundamental stage of adolescent development is the gradual transfer of decision making from parent to child that allows individuals to start taking responsibility for their own health (McCabe, 1996), and girls in this study were clearly at this stage. In consequence, had the vaccine decision rested only with them, the number of girls who might have initially refused or dropped out of the programme would have outweighed the number who would have opted or stayed in; hence, parental support for vaccination is still required.

Approximately 20% of the total study population of girls completed this questionnaire and their parents may have held more liberal views on adolescent participation in consent than those of nonresponders. Essentially the sample provides a good, but not necessarily typical, example of the mother–daughter interaction promoted by the Department of Health in its social marketing strategy to ‘arm against cancer’ (Department of Health, 2009b). The Department intends the literature to be read by mothers and daughters together, and is in line with previous observations that parents who talk to their daughters are more supportive of HPV vaccination (Brabin *et al*, 2006). This study suggests that it also helps girls to prioritise vaccination and think about future health and relationships (Table 1). Yet, some parents would like to defer HPV vaccination to a later age and avoid discussing sexual matters (Marlow *et al*, 2007; Woodhall *et al*, 2007). Having some sexual awareness, 21% of study girls already found the topic embarrassing and older girls would likely prefer to talk more with peers than with parents (Ogle *et al*, 2008). More directly worded questions on sexually transmitted infections would have allowed a better understanding of girls' awareness of sexual issues, but we decided against this, given the possibility of a parental veto. The national campaign could place more explicit emphasis on the importance of joint discussions and inform parents of the evidence that communication about sex with young adolescents positively influences their values in late (although not middle) teens (Fisher 1986). Nearly 80% of girls stated that the vaccine reminded them of the risks of sexual contact, and it would be instructive if future research were to show that HPV vaccination encouraged preventive actions rather than increased sexual risk taking.

Media coverage of reported serious adverse events attributed to HPV vaccination has followed vaccine introduction in several countries. Our report is the first to show that such rumours filter down to girls and become further exaggerated. One consequence is likely to be an increased reluctance to complete the three doses or to recommend the vaccine to younger sisters and friends. Parents also decline vaccination and find it stressful if their daughters dislike needles. (Rosenthal *et al*, 2008). In Australia, interim data for the school-based programme showed a fall in Dose 3 coverage in all territories and age groups (Brotherton *et al*, 2008). The third-dose uptake figure for the national vaccine programme in England will only be clear once all the missing doses have been followed up in the next academic year

### Conclusion

Parents still exert an important influence on girls at this stage of their development by talking to them, encouraging them to think about the importance of vaccination and having begun a vaccine course, to complete it. Without this, vaccine coverage could fall. Health professionals must address the misconceptions held by girls (and by some parents), thereby reducing uninformed discussions and helping girls to come to their own decision about HPV vaccination.

## Figures and Tables

**Table 1:** Number (%) of girls agreeing with statements about the implications of being vaccinated against a sexually transmitted HPV infection

**Statement**	**Number (%) agreeing**
I wouldn't tell a boyfriend I've been vaccinated against HPV	131 (24.8)
HPV injection is embarrassing because it's for a sexually transmitted infection	115 (21.4)
Having the vaccine shows that you are serious about your own health	514 (93.0)
Having the vaccination reminds me of the possible risks of sexual contact	420 (78.8)
I might take more risks in the future because I'm protected against HPV	73 (13.6)
Boyfriends may expect me to take more risks because I'm vaccinated.	99 (18.8)

Abbreviation: HPV=human papillomavirus.

## References

[bib1] Brabin L, Roberts RA, Farzaneh F, Kitchener HC (2006) Future acceptance of adolescent human papillomavirus vaccination: a survey of parental attitudes. Vaccine 24: 3087–3094 16500736 10.1016/j.vaccine.2006.01.048

[bib2] Brabin L, Roberts SA, Stretch R, Baxter D, Chambers G, Kitchener H, McCann R (2008) Uptake of first two doses of human papillomavirus vaccine by adolescent schoolgirls in Manchester: prospective cohort study. BMJ 336: 1056–1058 18436917 10.1136/bmj.39541.534109.BE PMC2375997

[bib3] Brotherton JM, Deeks SL, Campbell-Lloyd S, Misrachi A, Passaris I, Peterson K, Pitcher H, Scully M, Watson M, Webby R (2008) Interim estimates of human papillomavirus vaccination coverage in the school-based program in Australia. Commun Dis Intell 32: 457–461 10.33321/cdi.2008.32.45 19374275

[bib4] Department of Health (2009a) HPV Vaccination Programme: first and second dose vaccine uptake by January 2009 Provisional data. Accessible at: http://www.immunisation.nhs.uk/publications/HPV_uptake_data_April09.pdf

[bib5] Department of Health (2009b) HPV Campaign Press Adverts. Accessible at: http://www.immunisation.nhs.uk/Vaccines/HPV/Resources

[bib6] Fisher TD (1986) An exploratory study of parent-child communication about sex and the sexual attitudes of early, middle, and late adolescents. J Gen Pyschol 147: 543–557 10.1080/00221325.1986.9914529 3572373

[bib7] Marlow LA, Waller J, Wardle J (2007) Parental attitudes to pre-pubertal HPV vaccination. Vaccine 25: 1945–1952 17284337 10.1016/j.vaccine.2007.01.059

[bib8] McCabe MA (1996) Involving children and adolescents in medical decision-making: developmental and clinical considerations. J Ped Psychol 21: 505–516 10.1093/jpepsy/21.4.505 8863460

[bib9] Ogle S, Glasier A, Riley SC (2008) Communication between parents and their children about sexual health. Contraception 77: 283–288 18342652 10.1016/j.contraception.2007.12.003

[bib10] Rosenthal SL, Rupp R, Zimet GD, Meza HM, Loza ML, Short MB, Succop PA (2008) Uptake of HPV vaccine: demographics, sexual history and values, parenting style, and vaccine attitudes. J Adolesc Health 43: 239–245 18710678 10.1016/j.jadohealth.2008.06.009

[bib11] Stretch R, Chambers G, Whittaker J, Critchley T, Jackson F, Montgomery MB, Roberts S, Brabin L (2008a) Implementing a school-based HPV vaccination programme. Nurs Times 104: 30–33 19090363

[bib12] Stretch R, Roberts SA, McCann R, Baxter D, Chambers G, Kitchener H, Brabin L (2008b) Parental attitudes and information needs in an adolescent HPV vaccination programme. Br J Cancer 99: 1908–1911 18985038 10.1038/sj.bjc.6604766 PMC2600705

[bib13] Vallely L, Roberts S, Kitchener H, Brabin L (2008) Informing adolescents about human papillomavirus. Vaccine 26: 2203–2210 18394764 10.1016/j.vaccine.2008.02.055

[bib14] Woodhall SC, Lehtinen M, Verho T, Huhtala H, Hokkanen M, Kosunen E (2007) Anticipated acceptance of HPV vaccination at the baseline of implementation: a survey of parental and adolescent knowledge and attitudes in Finland. J Adol Health 40: 466–469 10.1016/j.jadohealth.2007.01.005 17448408

